# On the Feasibility of Stereotactic Radiosurgery With 5.0 and 10.0 mm MLC Leaves as a Function of Target Size and Shape

**DOI:** 10.3389/fonc.2019.00741

**Published:** 2019-08-07

**Authors:** Wassim Jalbout, Jana Abou Zahr, Bassem Youssef, Bilal Shahine

**Affiliations:** Radiation Oncology Department, American University of Beirut Medical Center, Beirut, Lebanon

**Keywords:** SRS, SRT, radiosurgery, MLC, multileaf collimators, leaf width, leaf size

## Abstract

**Introduction:** This study explores the feasibility of SRS/SRT treatment with MLC leaves wider than 2.5 mm at isocenter by inter-comparing treatment plans produced with 2.5, 5.0, and 10.0 mm leaves for various target sizes and shapes.

**Materials and methods:** Forty previously treated patients were re-planned using 2.5, 5.0, and 10.0 mm wide MLC leaves. For each patient, all three plans were evaluated and contrasted between them in terms of five metrics: target dose homogeneity, conformity index, organs at risk dose, dose fall off outside the target, and dose to normal tissues. A regularity index RI was introduced that quantified the degree of target shape irregularity. The effect of target size and shape irregularity on feasibility of 5.0 and 10.0 mm leaves was analyzed.

**Results:** Consistent plan degradation was observed for 10.0 mm (sometimes for 5.0 mm) compared to 2.5 mm MLC in terms of the above five plan metrics, but this degradation was small to clinically insignificant. As an exception, when target (PTV) size was smaller than about 1 cm diameter, clinically significant differences were found between 2.5, 5.0, and 10.0 mm MLC.

**Conclusion:** 5.0 and 10.0 mm MLC can be used in SRS/SRT for targets (PTV) diameter larger than 1 cm. For smaller targets, 2.5 mm MLC is clinically superior, 5.0 is acceptable and 10.0 mm MLC is discouraged in terms of PTV dose conformity.

## Introduction

Stereotactic radiosurgery/radiotherapy (SRS/SRT) treatment techniques are now essential components of most modern radiotherapy departments' treatment modalities. Their application is very common in the treatment of cranial metastases and is becoming progressively more so with recent indications favoring SRS/SRT over whole brain irradiation in certain cases of multiple brain metastasis.

Historically, when most linacs had either no multileaf collimator (MLC) or 10.0 mm MLC leaf width (leaf width is specified at isocenter throughout this text), SRS/SRT treatments were carried out using circular cones or an external add-on MLC with 2.5 to 3.0 mm leaf size. With the availability of integrated native 5.0 mm leaf MLC in modern linacs, many centers abandoned the add-on MLC solution in favor of the native 5.0 mm MLC with acceptable clinical results.

However, some centers (mostly in developing countries) are still operating linacs with 10.0 mm MLC leaves, and the question arises as to whether it is acceptable to perform SRS/SRT treatments at such a leaf width.

The advantage of small leaf width in SRS/SRT treatments has been studied by several groups (1–5), but with mixed results. According to Monk et al. (1) in a (small) study of 14 intracranial cases using BrainSCAN v5.1 for re-planning, 3 mm MLC leaves improved both target conformity and normal tissue sparing over 5.0 mm leaves but these improvements were not very significant clinically; also no statistically significant differences were found in the maximum dose to critical structures.

Chern et al. (2) studied 23 patients previously treated for intracranial lesions using BrainSCAN version 5.3 to perform a dosimetric comparison of 3 mm leaf width microMLC and 5.0 mm MLC. They too found very small advantages to the 3.0 mm leaves and of questionable clinical significance.

Others studied the dosimetric impacts of different leaf widths across various treatment techniques. Wu et al. (3), compared treatment plans with 2.5 mm leaves and 5.0 mm leaves, for different treatment techniques and for a subset of five brain tumor cases abutting the brainstem. They concluded that the 2.5 mm leaves in combination with the IMRT technique can yield small dosimetric benefits (over 5.0 mm leaves) to the treatment of small lesions in cases involving irregular target shapes or organ-at-risk shapes, but they questioned the clinical significance of these small differences. Tanyi et al. (4) reported similar results.

Santos et al. (5) evaluated the possibility of performing SRS with 5.0 mm leaves instead of 3.0 mm. They investigated 90 treated patients, where they re-planned each one with both leaves on iPlan version 4.5 from Brainlab. The plan quality evaluation parameters were target coverage, dose conformity, conformity gradient index, minimum and maximum target doses, dose to critical structures and dose to normal tissue. They concluded that the most affected parameters were dose to critical structures located close to target and target dose conformity for irregular targets. However, two confounding factors exist in their study: first, the 3.0 mm leaves plan consisted in dynamic conformal arcs while the 5.0 mm leaves plan consisted in non-dynamic conformal arcs; this difference may confer higher dose conformity to the 3.0 mm plan regardless of leaf width. Second, they did not equalize target coverage in both plans before comparing organ at risk doses (it is easy to greatly spare an organ at risk by accepting target under-coverage).

In this work, we propose a regularity index RI that quantifies target shape irregularity and we attempt to provide numerical limits for target dimension and irregularity within which 5.0 and 10.0 mm MLC could be acceptable for SRS/SRT treatments instead of 2.5 mm.

## Methods and Materials

### Patient Sample and Equipment

Ethics approval for the study was not required by local legislation and laws as the data being used was de-identified patient data. A set of forty patients previously treated for cranial SRS/SRT in the radiation therapy department of the American University of Beirut Medical Center, Beirut, Lebanon between 2016 and 2018, constitute the basis of this study. The selected patients had lesions of different sizes and degrees of shape irregularity, and most of them were located close to critical structures. Patients were from all age groups, and the SRS/SRT treatment they had received in our center differed in terms of total prescribed dose, number of fractions, and number of beams. Patient details as originally treated are summarized in Table 1, which shows that a relatively wide variety of intracranial cases were covered from single fraction SRS to standard fractionated SRT.

**Table 1:** Patients and treatment plan characteristics as originally treated.

	**Age**	**Prescribed dose (Gy)**	**No. of fractions**	**Prescription isodose (%)**	**No. of beams**	**MLC margin (mm)**	**PTV volume (cc)**
**Range**	14 – 80	10 – 52.2	1 – 29	77 – 100	8 – 18	0 – 5	0.332 – 98.025
**Average**	58	25	5	91	14	2	16

**Figure 1:**
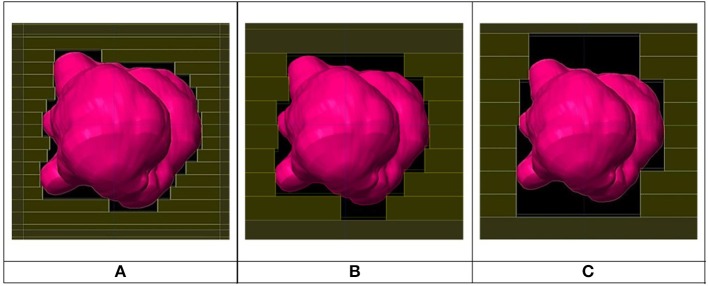
Beam's eye view for same beam for 2.5 mm **(A)**, 5.0 mm **(B)**, and 10.0 mm **(C)** leaf width MLCs.

Three plans were created for each of the forty patients, one with 2.5 mm leaf width, one with 5.0 mm and another with 10.0 mm and all plans were of the forward type. Figure 1 shows the Beam's Eye View for three different leaf sizes. The IMRS technique (Intensity Modulated RadioSurgery) was not used due to lack of availability. All planning was done using treatment planning system Brainlab's iPlan v.4.5 (Brainlab AG, Germany) with a Siemens Artiste linac (Siemens, Germany) commissioned into iPlan. The 2.5 mm leaf plans used an add-on external 80 leaves microMLC (Moduleaf, by Siemens) commissioned into iPlan. The 5.0 mm plans used the linac's integrated native 160 leaves 5.0 mm MLC (replacing the X jaws) commissioned into iPlan. To create plans with 10.0 mm MLC leaf width in the absence of an available physical 10.0 mm leaf MLC, the 5.0 mm MLC was used with each two adjacent leaves grouped as one by manual adjustment in Beam's Eye View mode (Figure 1C). All plans used conformal static beams (8–18 beams for each plan) as the rotational dynamic MLC technique was not available.

The following parameters were made identical for the above three plans: number of beams, beam weights, beam and couch angles, margin around PTV, calculation algorithm used (Pencil Beam), 1 mm dose grid resolution and beam energy (7 MV unflat @ 2,000 MU/min). However, the collimator angle was individually optimized for each field in each plan for best dose conformity around the target. For the 2.5 and 5.0 mm leaves, collimator angle optimization was automatically provided by iPlan; for the 10.0 mm leaf plan, optimization of collimator rotation was done manually and visually in Beam's Eye View mode by orienting the leaves perpendicularly to the long sides of the target, as well as by centering the leaves facing a PTV concavity so they can slide into it as deep as possible.

Original plan prescriptions were all changed to a single prescription dose of 20 Gy and all plans were normalized so that the 20 Gy isodose cloud covered 95% of the PTV volume. Imposing equal target coverage by the prescription isodose across all plans was necessary to ensure fairness in plan comparison.

### Plan Merit Evaluation Metrics

#### Dose Conformity Index

Conformity was measured by a conformity index CI following Paddick's equation:

(1) $$\begin{array}{l} CI=\;\frac{V_{\cap }}{V_{PTV}}\;\times \frac{V_{\cap }}{V_{isod}} \end{array}$$

where

V_PTV_: PTV volume

V_isod_: Volume of prescription isodose cloud (20 Gy cloud)

V_∩_: V_PTV_ ∩ V_isod_

**Figure 2:**
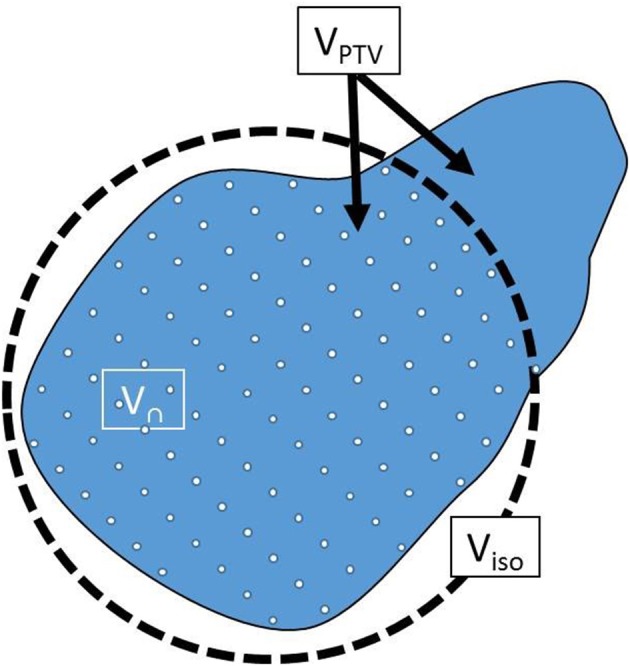
Illustration of V_n_, V_PTV_, and V_isod_ used in Equation (1).

V_∩_, V_PTV_ and V_isod_ are further illustrated in Figure 2.

The first ratio in Equation (1) represented PTV coverage, forced in this work to a value of 95% in all plans by appropriate normalization. The second ratio measures over-coverage of PTV by the isodose cloud; it is usually <1.0 and optimal at a value of 1.0. Therefore, the higher the CI the higher the dose conformity and vice versa.

**Figure 3:**
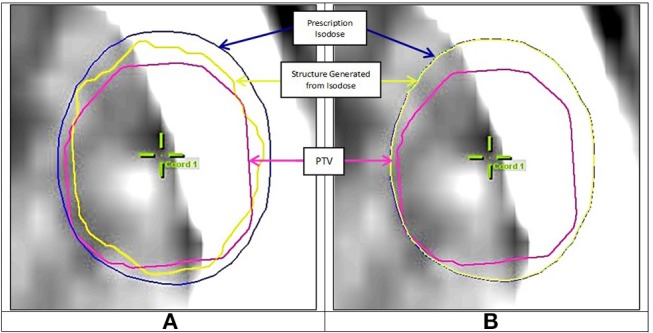
**(A)** Mismatch between prescription isodose and iPlan generated structure, **(B)** Good match when using Velocity AI.

The evaluation of terms V_isod_ and V_∩_ in Equation (1) required first the transformation of an isodose cloud into a structure, then getting its volume. It was observed that the structure iPlan created out of the isodose cloud did not match the isodose cloud itself in the case of small PTVs. This occurred because iPlan uses different calculation grid parameters for isodose calculation vs. structure calculation (Personal communication with BrainLAB Company). All plans were therefore exported into software Velocity AI (Varian, USA) where this problem was not encountered. Figure 3A shows the prescription isodose and the structure generated from iPlan. A mismatch can be seen between the generated dose structure and the isodose it is supposed to match with. However, Figure 3B shows a good match when using Velocity AI. Therefore, numerical values were obtained from Velocity AI for all volumes in Equation (1), then CI was calculated manually.

#### Target Shape Regularity

To quantify the degree of target shape regularity, we proposed a regularity index (RI) that compares the PTV's shape to that of a sphere, with RI defined as follows:

(2) $$\begin{array}{l} RI=\;\frac{S_{eq\;sph}}{S_{PTV}} \end{array}$$

where

S_eq_ _sph_ is the Surface area of a sphere having same volume as the PTV, and

S_PTV_ is the actual PTV's surface

**Figure 4:**
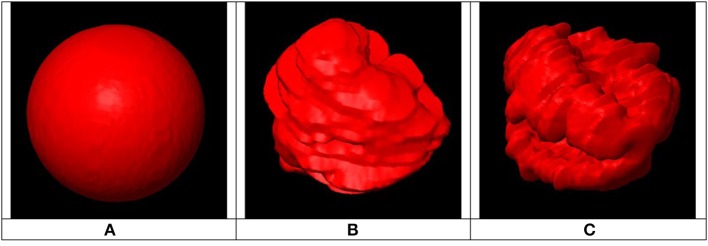
Example of three PTVs with same volumes, different surface areas and different regularity indices: **(A)** Smallest surface area (RI = 1) associated with highly regular shape, **(B)** Medium shape regularity (RI < 1), **(C)** Largest surface area (RI << 1) associated with highly irregular shape.

This index was proposed because a sphere is a 3D structure with optimal shape regularity and because shape regularity is directly linked to surface area; the higher a structure's shape irregularity, the higher its surface area. To allow a comparison of PTV shape regularity to a sphere, both must have the same volume. The higher the value of RI in Equation (2), the higher the target shape's regularity, with a maximum value of 1.0 reached when the PTV is perfectly spherical. This is illustrated in Figure 4 where three different targets are shown having same volume but different shape regularity associated with different RI values.

S_eq_ _sph_ was obtained by first calculating the radius r_eq_ of a sphere of equal volume to the PTV:

(3) $$\begin{array}{l} r_{eq}=\sqrt[{3}]{\frac{3\;V_{PTV}}{4\;\pi }} \end{array}$$

Then S_eq_ _sph_ was calculated as:

(4) $$\begin{array}{l} S_{eq\;sph}=4\;\pi \;r_{eq}^{2} \end{array}$$

Since iPlan did not provide a value for S_PTV_, it was obtained by importing the PTV into 3D Slicer software (3D Slicer, RRID:SCR_005619) (6, 7) then extracting its surface area.

#### Target Dose Homogeneity and Organs at Risk Dose

Target dose homogeneity and organs at risk dose were both analyzed in terms of Dmin, Dmean, and Dmax obtained directly from iPlan treatment planning system.

#### Dose Fall Off

**Figure 5:**
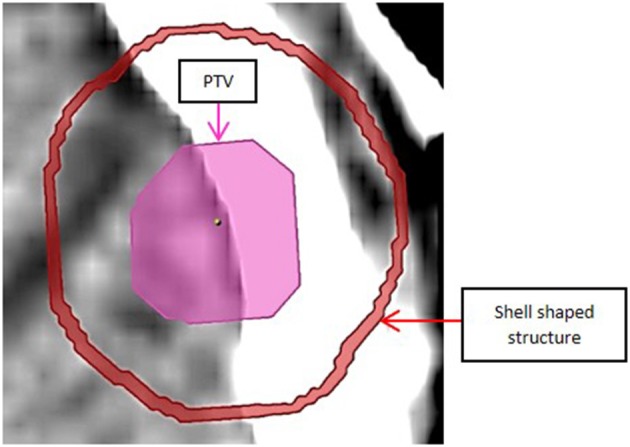
Shell shaped structure located 5.0 mm away from PTV surface to estimate dose fall off.

To estimate dose fall off outside the target, a 1 mm thick shell shaped volume was generated 5 mm away from the PTV surface and around it, as shown in Figure 5, and Dmean and Dmax for the shell (obtained from iPlan) were compared between plans of different leaf thickness.

#### Normal Tissue Dose

Finally, to analyze normal tissue dose, normal tissue was defined as difference between cranium outer contour and planning target volume. Normal tissue dose was analyzed in terms of Dmean and Dmax obtained from iPlan.

## Results and Discussion

### Target Dose Homogeneity

Table 2 below gives minimum, mean, and maximum dose values for the PTV (Dmin, Dmean, and Dmax) averaged over all patients for each leaf width. Dmin, Dmean, and Dmax are all expressed in percent of prescription dose.

**Table 2:** PTV coverage for each of the three MLC leaf sizes.

	**PTV Coverage**								
	**Dmin (%)**			**Dmean (%)**			**Dmax (%)**		
	**2.5 mm**	**5.0 mm**	**10.0 mm**	**2.5 mm**	**5.0 mm**	**10.0 mm**	**2.5 mm**	**5.0 mm**	**10.0 mm**
Mean	79.6	79.8	81.3	97.4	97.7	97.0	102.5	103.2	102.0
SD	17.1	15.6	15.5	4.8	4.9	5.3	5.3	5.1	5.8

Based on Table 2, leaf width does not have a clinically significant impact on dose homogeneity inside the PTV.

### Target Dose Conformity

#### As a Function of Target Size

**Figure 6:**
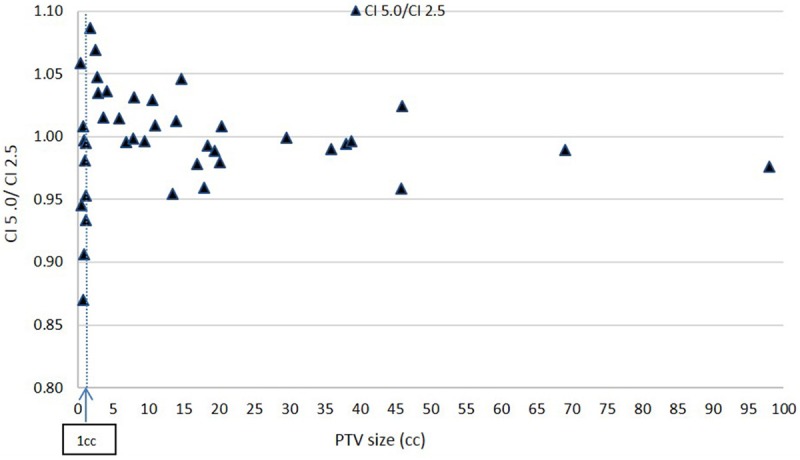
Ratio of Conformity Indices CI 5.0 to CI 2.5 as a function of PTV size.

**Figure 7:**
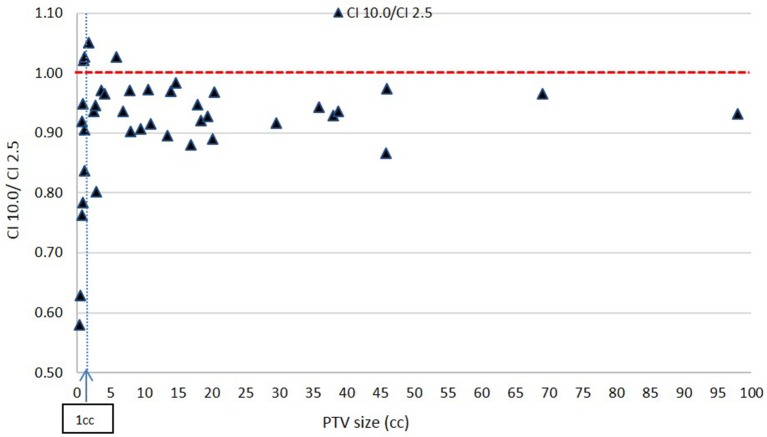
Ratio of Conformity Indices CI 10.0 to CI 2.5 as a function of PTV size.

Figures 6, 7 show plots of the ratios of Conformity Indices per equation 1, respectively, CI 5.0/CI 2.5 and CI 10.0/CI 2.5 for all patients as a function of PTV size in cc.

Based on Figure 6, there is no clear inferiority of CI 5.0 to CI 2.5 mm when PTV is larger than about 1 cc as data points are both above and below the horizontal 1.00 ratio line. But for PTV volume smaller than about 1 cc, CI 5.0 mm is generally smaller than CI 2.5 mm indicating a degradation in dose conformity with 5.0 mm leaves. This disadvantage of 5.0 mm MLC is illustrated in Figure 1 where the ladder shaped spaces at leaf end outside of the PTV are larger with 5.0 mm leaves compared to 2.5 mm leaves.

Still, for such small targets, the loss of conformity from 5.0 mm leaves may be forgiven since the additional volume unintentionally receiving treatment around the PTV may be considered small, thus clinically acceptable, based on a maximum of 15% decrease in CI from 2.5 to 5.0 mm leaves from Figure 6 at very small field sizes.

Figure 7 shows a consistent inferiority in conformity for the 10.0 mm MLC compared to the 2.5 mm MLC across all PTV sizes. This result is expected. While majority of these decreases in CI value with the 10.0 mm leaves are within 10% and may be considered clinically acceptable, the figure shows a prohibitively large decrease in CI ratios at very small PTVs: for PTV size smaller than about 1 cc (in the order of 1 cm diameter), CI values for the 10.0 mm leaves reach a value close to 0.57, which makes the 10.0 mm leaves not recommendable for clinical usage.

#### As a Function of Target Shape

**Figure 8:**
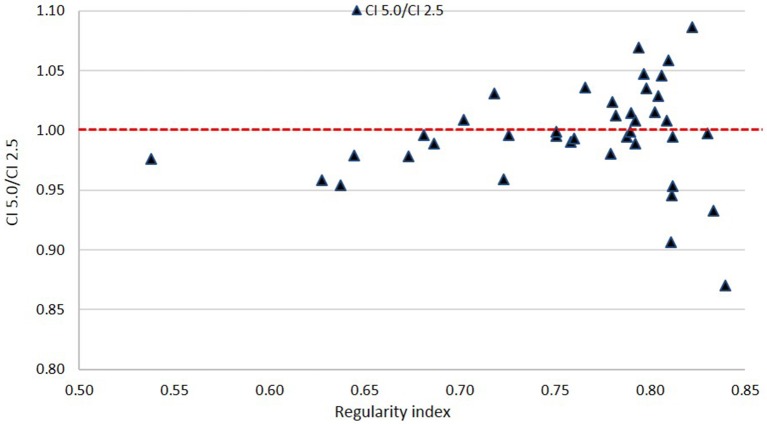
Ratio of Conformity Indices CI 5.0 to CI 2.5 as a function of Regularity Index.

**Figure 9:**
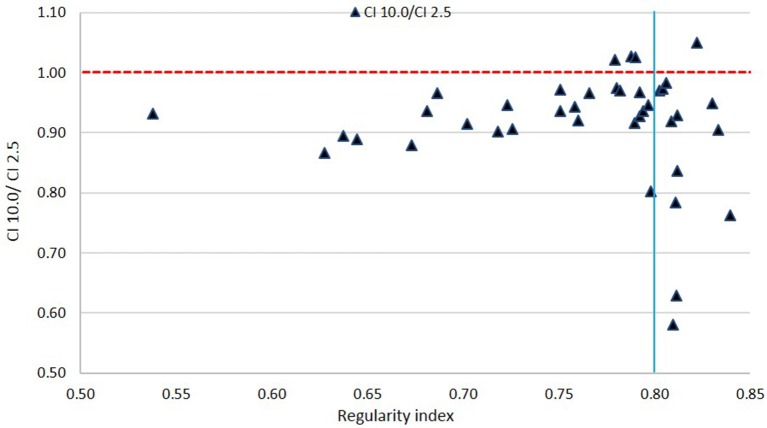
Ratio of Conformity Indices CI 10.0 to CI 2.5 as a function of Regularity Index. The vertical line marks the 0.80 RI value.

Figures 8, 9 show plots of the ratios of Conformity Indices per equation 1, respectively, CI 5.0/CI 2.5 and CI 10.0/CI 2.5 for all patients as a function of PTV shape regularity.

Based on Figure 8, there is no clear disadvantage to the 5.0 mm leaves compared to the 2.5 mm leaves across all values of RI. Scores are somewhat evenly distributed above and below the 1.00 ratio line. This suggests that there is no specific cutoff value for PTV shape regularity below which use of 5.0 mm leaves becomes problematic.

In Figure 9, a general inferiority in dose conformity (within 10% for the most part) is seen for 10.0 mm leaves to 2.5 mm leaves across most RI values. A cluster of very low conformity points with 10 mm MLC is seen around 0.80 RI ratio; these represent PTVs with regular shapes (RI is high) but happen to be small in size, and the conformity loss with 10.0 mm leaves here is due to the small PTV size as mentioned above as seen in Figure 7.

### Organs at Risk Dose

Table 3 a–f gives values of maximum dose (Dmax) in Gy to six organs at risk, averaged over all 40 patients for all three MLC leaf sizes. Again, all plans were normalized to a prescription of 20 Gy to allow for a consistent comparison.

**Table 3:** Maximum dose to organs at risk for a prescription dose of 20 Gy.

**(a)**	**Brainstem**			**(b)**	**Left optic nerve**		
Leaf size, mm	2.5	5.0	10.0	Leaf size, mm	2.5	5.0	10.0
Dmax (Gy) averaged over all patients	9.3	9.7	10	Dmax (Gy) averaged over all patients	3.2	3.4	3.5
SD	9.1	9.1	9.1	SD	6.3	6.3	6.3
**(c)**	**Optic chiasm**			**(d)**	**Right eye**		
Leaf size, mm	2.5	5.0	10.0	Leaf size, mm	2.5	5.0	10.0
Dmax (Gy) averaged over all patients	4.3	4.5	4.8	Dmax (Gy) averaged over all patients	0.7	0.8	0.9
SD	7.1	7.2	7.2	SD	0.2	0.3	0.5
**(e)**	**Right optic nerve**			**(f)**	**Left eye**		
Leaf size, mm	2.5	5.0	10.0	Leaf size, mm	2.5	5.0	10.0
Dmax (Gy) averaged over all patients	3.5	3.6	3.7	Dmax (Gy) averaged over all patients	0.7	0.8	1
SD	6.6	6.6	6.7	SD	1.1	1.2	1.5

In general no clinically significant differences could be seen between different leaf sizes. In cases where the organ at risk was within 2 cm from PTV, we could see differences between different MLCs of about 1 Gy (This is not shown in Table 3).

### Dose Fall Off

Table 4 gives mean dose (Dmean) and maximum dose (Dmax) (as % of 20 Gy prescription dose) for the spherical shell shaped volume located 5 mm away from and around the PTV, defined in Figure 5, averaged over all patients.

**Table 4:** Dose fall-off at 5 mm from PTV for each of the three MLC leaf sizes.

	**Dmean (%)**			**Dmax (%)**		
Leaf size, mm	2.5	5.0	10.0	2.5	5.0	10.0
Averaged over all patients	46.1	52.1	56.7	74.6	75.9	80.7
SD	11.1	10.3	11	14.4	12.3	11.6

Data in Table 4 shows a superiority of the 2.5 mm leaves in steepness of dose fall off compared to 5.0 and 10.0 mm leaves. In terms of mean dose Dmean, the difference between the 2.5 mm leaves and the 5.0 or 10.0 mm leaves is about 6–10% of the prescription dose or 1.2–2 Gy. In terms of maximum dose Dmax, the difference between the 2.5 and the 5.0 mm leaves is very small and the difference between the 2.5 mm and the 10.0 mm leaves is 1.2 Gy. Of the two dosimetric quantities used to analyze dose fall-off, Dmax appears more relevant than Dmean since the organs at risk at hand in cranial SRS treatments (brainstem, optics) have limit doses better defined in terms of Dmax than Dmean. Differences in Dmax falloff of around 1.2 Gy between 2.5 and 10.0 mm may be clinically acceptable, depending on the particular case.

The dose calculation was performed here with Pencil Beam algorithm. Although more advanced algorithms may describe dose fall off more accurately, Pencil Beam should be adequate here since we are only comparing fall off rates.

### Normal Tissue Dose

Normal tissue volume was defined as external cranial contour minus PTV. Both mean dose and maximum dose were scored for the normal tissue volume and are presented in Table 5 for each MLC leaf size. An increase in mean dose, albeit slow and of questionable clinical significance, can be seen with increase in leaf size.

**Table 5:** Dose to normal tissue for each of the three MLC leaf sizes expressed in % of prescription dose.

	**Normal Tissue**					
	**Mean Dose (%)**			**Max Dose (%)**		
	**2.5 mm**	**5.0 mm**	**10.0 mm**	**2.5 mm**	**5.0 mm**	**10.0 mm**
Mean	4.53	4.90	5.67	97.60	98.02	97.86
SD	3.34	3.56	3.78	6.43	6.35	6.49

## Conclusion

Forty SRT/SRS patients were planned with 2.5, 5.0, and 10.0 mm MLC leaves and the plans were compared and analyzed to determine the validity of 5.0 and 10.0 mm MLC as a function of PTV size and shape.

No clinically relevant difference was found between different leaf sizes in terms of target dose homogeneity.

In terms of target dose conformity, 2.5 mm leaves did not give consistently better results than 5.0 mm, but they did give consistently better results than 10.0 mm leaves. Despite their inferiority to the 2.5 mm leaves, the 10.0 mm leaves produced generally clinically acceptable results for PTVs larger than about 1.0 cc (or about 1.0 cm diameter). But for PTVs smaller than 1.0 cm diameter, 10.0 mm leaves produced relatively bad dose conformity results. PTV shape regularity did not seem to influence dose conformity differences between the three leaves in a clinically relevant manner. Although this last result seems counter intuitive since smaller leaves conform better to the PTV's surface irregularities, it could be that the PTV shapes encountered clinically are generally not irregular enough for a measurable advantage to the 2.5 mm leaves to be detected over 5.0 and 10.0 mm.

In terms of organ at risk dose and normal tissue dose, no clinically determining differences were found between the three leaf sizes.

In terms of dose falloff, a disadvantage was found to the 5.0 and 10.0 mm leaves compared to 2.5 mm leaves; however this difference seemed clinically acceptable.

Therefore, this work suggests that 5.0 mm leaves are a clinically acceptable alternative to 2.5 mm leaves in all the cases covered here. 10.0 mm leaves also can be used in SRS/SRT as long as PTV diameter remains larger than about 1.0 cm.

For these conclusions to be clinically applicable, further studies are required.

## Data Availability

All datasets generated for this study are included in the manuscript/supplementary files.

## Author Contributions

WJ worked with JA to generate the data and write the paper and supervised the work. JA worked with WJ to generate the data and write the paper. BY and BS contributed equally by providing the paper's concept and guidance throughout.

### Conflict of Interest Statement

The authors declare that the research was conducted in the absence of any commercial or financial relationships that could be construed as a potential conflict of interest.
